# The *Drosophila *Anion Exchanger (DAE) lacks a detectable interaction with the spectrin cytoskeleton

**DOI:** 10.1186/1477-5751-9-5

**Published:** 2010-06-23

**Authors:** Ronald R Dubreuil, Amlan Das, Christine Base, G Harper Mazock

**Affiliations:** 1 Dept. of Biological Sciences, University of Illinois at Chicago, 900 S. Ashland Ave., Chicago, IL 60607 USA; 2 433 S. University Ave., Lynch Laboratories, Dept. of Biology, University of Pennsylvania, Philadelphia, PA 19104 USA

## Abstract

**Background:**

Current models suggest that the spectrin cytoskeleton stabilizes interacting ion transport proteins at the plasma membrane. The human erythrocyte anion exchanger (AE1) was the first membrane transport protein found to be associated with the spectrin cytoskeleton. Here we evaluated a conserved anion exchanger from Drosophila (DAE) as a marker for studies of the downstream effects of spectrin cytoskeleton mutations.

**Results:**

Sequence comparisons established that DAE belongs to the SLC4A1-3 subfamily of anion exchangers that includes human AE1. Striking sequence conservation was observed in the C-terminal membrane transport domain and parts of the N-terminal cytoplasmic domain, but not in the proposed ankyrin-binding site. Using an antibody raised against DAE and a recombinant transgene expressed in *Drosophila *S2 cells DAE was shown to be a 136 kd plasma membrane protein. A major site of expression was found in the stomach acid-secreting region of the larval midgut. DAE codistributed with an infolded subcompartment of the basal plasma membrane of interstitial cells. However, spectrin did not codistribute with DAE at this site or in anterior midgut cells that abundantly expressed both spectrin and DAE. Ubiquitous knockdown of DAE with dsRNA eliminated antibody staining and was lethal, indicating that DAE is an essential gene product in *Drosophila*.

**Conclusions:**

Based on the lack of colocalization and the lack of sequence conservation at the ankyrin-binding site, it appears that the well-characterized interaction between AE1 and the spectrin cytoskeleton in erythrocytes is not conserved in *Drosophila*. The results establish a pattern in which most of the known interactions between the spectrin cytoskeleton and the plasma membrane in mammals do not appear to be conserved in *Drosophila*.

## Background

The spectrin cytoskeleton forms a submembrane protein scaffold that contributes to cell shape and membrane stability in the human erythrocyte [reviewed in [1]]. Biochemical studies identified the anion exchanger as the primary membrane anchor that attaches the spectrin cytoskeleton to the erythrocyte plasma membrane. Attachment is mediated by the protein ankyrin which serves as an adapter linking the N-terminal cytoplasmic domain of the anion exchanger to the β subunit of erythrocyte spectrin [2].

Subsequent studies of the spectrin cytoskeleton in more complex cells have uncovered a remarkable diversity of different membrane proteins attached to ankyrin. Many of these are physiologically important transporters and channels whose distribution in the cell is critical to function [3,4]. Most of these integral membrane proteins appear to rely on their interaction with the spectrin cytoskeleton to be stably expressed at the cell surface. Consequently, mutations that knock out or inactivate ankyrin or spectrin lead to a dramatic reduction in their steady-state levels.

Spectrins and ankyrins are conserved between humans and *Drosophila*. There is a single conventional spectrin in *Drosophila *that is composed of α and β subunits arranged as a tetramer. *Drosophila *spectrin is nearly indistinguishable from human spectrin by electron microscopy, it possesses most of the known functional sites (e.g. actin-binding, ankyrin-binding, intersubunit interactions, PH domain, etc.) and it is found associated with the plasma membrane in most *Drosophila *cells that have been examined [5]. Ankyrin is also conserved between *Drosophila *and humans. Ankyrins possess an N-terminal membrane binding domain composed of ankyrin repeats and a central spectrin binding domain. The two isoforms of ankyrin in *Drosophila *are similar to one another in their N-terminal and spectrin-binding domains, but their C-terminal domains are different, with further diversity added by alternative splicing of the neuronal ankyrin isoform DAnk2 [6-8]. Interestingly, there is comparable sequence diversity between mammalian ankyrin isoforms in the C-terminal domain with only limited similarity to *Drosophila *ankyrins [2,7].

Yet, while spectrin and ankyrin are conserved in *Drosophila*, a remarkable divergence has become apparent in recent studies of candidate membrane anchors. Out of five interactions that have been examined so far only the interaction with L1 family cell adhesion molecules and ankyrin appears to be conserved in *Drosophila*. The L1 family member neuroglian possesses a conserved ankyrin-binding sequence in its cytoplasmic domain and it exhibits a functional interaction with ankyrin as well [9]. Another cell adhesion molecule, E-cadherin, was recently shown to interact directly with ankyrin in mammals [10]. In contrast, DE-cadherin (the fly counterpart of E-cadherin) does not appear to interact with ankyrin in *Drosophila *[11]. Two other ankyrin-binding membrane proteins in mammals, voltage-dependent sodium channels and KCNQ potassium channels, are conserved in their transmembrane ion-conducting domains but the domains responsible for binding to ankyrin in humans are not conserved in *Drosophila *[12]. The Na, K ATPase appears to be functionally linked to spectrin in *Drosophila*, since its behavior is altered in β spectrin mutants [13]. However, while the connection appears to be mediated by ankyrin in mammals [14], ankyrin-binding activity does not appear to be required for the effect of spectrin on the Na, K ATPase in *Drosophila *[15].

To expand the repertoire of membrane proteins that can be analyzed, we took advantage of molecular tools generated by the *Drosophila *genome project. A homolog of the erythrocyte anion exchanger was identified in the genomic sequence of *Drosophila*. It was an attractive candidate for further analysis because of its well-known interaction with ankyrin in mammals. The anion exchanger belongs to a family of closely related genes (AE1, AE2 and AE3; also known as SLC4A1-3) and to a larger family of 10 related genes that transport bicarbonate (SLC4 A1-10 [ref. [16,17]]). A conserved bicarbonate transporter (NDAE1) was previously identified in *Drosophila *and was shown to resemble the human sodium-dependent anion exchanger SLC4A8 [18]. Here we describe the properties of a second *Drosophila *anion exchanger (DAE) that it is closely related to human SLC4A1-3. Based on sequence comparisons and protein localization experiments, it appears that the well-characterized interaction between AE1 and the spectrin cytoskeleton in human erythrocytes is not conserved in *Drosophila*.

## Results

### Amino acid sequence analysis

**Figure 1:**
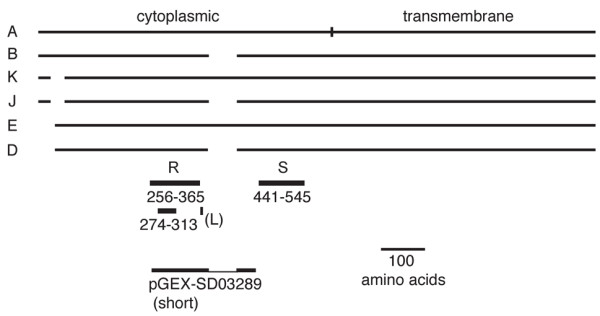
**Alternate protein products of the *Drosophila *anion exchanger gene**. The six major classes of DAE protein products are depicted relative to the longest class (A). Classes K and J use an alternate splice site that removes part of the coding sequence near the N-terminus. Classes E and D use an alternate transcription start site and an internal methionine start codon. Classes B, J and D splice out an alternate exon which is located between two zones with high sequence identity (R and S). The short loop sequence (L) which is responsible for the interaction between AE1 and ankyrin in humans is not conserved in DAE. The boundaries of a short recombinant fragment of DAE expressed as a pGEX fusion protein are also shown.

A *Drosophila *anion exchanger (DAE) related to mammalian AE1 was first identified among expressed sequence tags (ESTs) from the *Drosophila *genome project [19]. Analysis of data in FlyBase [20] indicates that there are 6 major polypeptide classes, shown relative to the longest class (A) in Figure 1, which are encoded by a number of different mRNAs. Classes E and D use an alternate 5' exon and an internal start methionine relative to A. Classes K and J use an alternate splice acceptor site, leading to deletion of 35 codons. Classes B, D and J splice out an alternate exon, leading to deletion of 67 codons. We used the amino acid sequence of clone RE39419 ([ref. [21]]; class B) for all of the work reported here. The sequence differences between classes in the N-terminal coding region occur within a region that is poorly conserved among AE family members. Likewise, the sequence of the skipped exon, absent from class B, did not match the sequence of other known anion exchangers.

Sequence comparisons established that the *Drosophila *anion exchanger belongs to the AE subfamily that includes mammalian AE1, AE2 and AE3 (Table 1). This subfamily represents the Na^+^-independent, electro-neutral anion exchangers. DAE shares 43 - 45% sequence identity with human AE1, 2 and 3, (Table 1) but only 26 - 40% identity with the other 7 members of the SLC4A gene family (not shown), including NDAE1. Thus DAE and NDAE1 appear to be in distinct anion exchanger subfamilies.

**Table 1:** Amino acid sequence comparison of human and Drosophila anion exchangers (% identity)

	NDAE1	DAE	AE1	AE2	AE3
NDAE1	-	40	38	40	40
DAE		-	45	43	44
AE1			-	60	57
AE2				-	62
AE3					-

**Figure 2:**
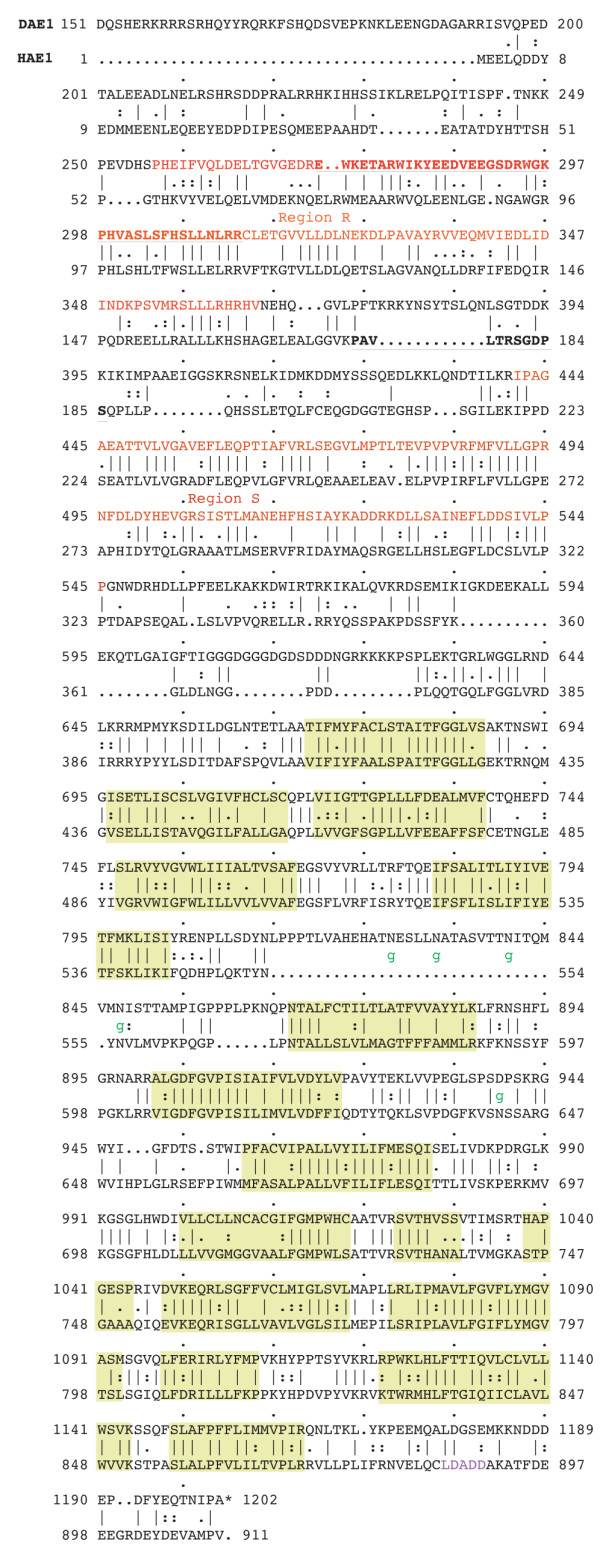
**Amino acid sequence alignment between DAE and human erythrocyte AE1**. The positions of 16 predicted transmembrane sequences are indicated in green boxes. The boundaries of the conserved cytoplasmic domain sequences R and S are indicated in red. The conserved subregion R' is indicated in bold red type. The sequence of the ankyrin-binding site in human AE1 is indicated in bold black type. Consensus glycosylation sites in the linker between transmembrane regions [5,6] and [7,8] are each marked by g. The site of interaction between the C-terminal domain of AE1 and carbonic anhydrase is indicated in purple.

Sequence alignments highlighted a number of features of the DAE sequence. First, the overall domain structure of DAE is essentially identical to human erythrocyte AE1 (Figure 2). A large N-terminal domain, ending with amino acid 667 of DAE, corresponds to the large N-terminal cytoplasmic domain of AE1. From that point on there is a close register between the sequences of human AE1 and DAE, corresponding to the position of transmembrane sequences (highlighted in green) and intra- and extra-cellular loops [22]. The only length variations occur in the two large extracellular loops between transmembrane domains 5 and 6 [TM5-6] and between [TM7-8]. The distribution of predicted glycosylation sites was not conserved between human AE1 and DAE. There are 4 N-linked glycosylation consensus sites (NxS/T) in the [TM5-6] linker of DAE (indicated by "g"), but none in human AE1 (Figure 2). There is a single conserved glycosylation site in the [TM7-8] linker of AE1 ("g"), but none in DAE. Like DAE, human AE2 had no consensus glycosylation sites in the [TM7-8] linker, one consensus site was shared with DAE in linker [TM5-6] and there were two other consensus sites in that linker that were not found in DAE. Thus there was a greater correspondence in predicted glycosylation patterns between AE2 and DAE than between AE2 and AE1.

Sequence comparisons also revealed several notable features of the N-terminal and C-terminal cytoplasmic domains of DAE. Two regions of substantial sequence identity near the N-terminus (R and S, highlighted in red in Figure 2) were sandwiched between three domains of limited sequence identity. Some of this sequence conservation coincides with the cytoplasmic domain pH sensor of mammalian AE2 (region R [ref. [17]]). Sequence comparisons in this region established a hierarchy of sequence identities with the highest between human AE2 and AE3, the next highest between DAE and AE2/3, and the lowest between AE1 and the others. A 40 residue sequence within the first conserved region (R'; bold letters) has been noted previously for its conservation among anion exchangers [17,23] and for its role in pH regulation of AE2 and AE3, but not AE1 [24]. The R' sequence was nearly identical between AE2 and AE3, DAE was 80% identical to AE2 in this region, and AE1 was 53% identical to AE2. The other conserved region (S) exhibited the same overall pattern of sequence identities, further demonstrating divergence of AE1 relative to DAE.

Recent structural studies mapped an ankyrin interaction site within an 11 amino acid loop in the N-terminal cytoplasmic domain of AE1 (L in Figure 1; bold black characters in Figure 2 [ref. [25]]). That loop falls in between the zones of sequence identity described above. In fact, the sequence alignment between DAE and AE1 inserted a gap precisely at that site, because of the limited sequence homology between the two proteins in between regions A and B. Gaps were also introduced at this site in comparisons between human AE1, AE2 and AE3, suggesting that this binding site is not a conserved feature of the AE gene family.

The sequence LDADD near the C-terminus of AE1 (purple text) is thought to be responsible for a functional interaction between carbonic anhydrase and mammalian anion exchangers [26]. A similar sequence was present in AE2 (LDANE), AE3 (LDSED), and in DAE (LDGSE), but not in NDAE1 (LDDIM). This pattern of sequence conservation further supports the grouping of DAE within the AE subfamily of anion exchangers.

### Production and characterization of a DAE antibody

A polyclonal antiserum was produced in rabbits using a purified glutathione transferase fusion protein containing 140 amino acids from the cytoplasmic domain of DAE. The resulting antibody produced a robust response in western blots of the recombinant fusion protein (not shown). The antibody was affinity-purified before further use and cross-adsorbed with purified glutathione transferase.

**Figure 3:**
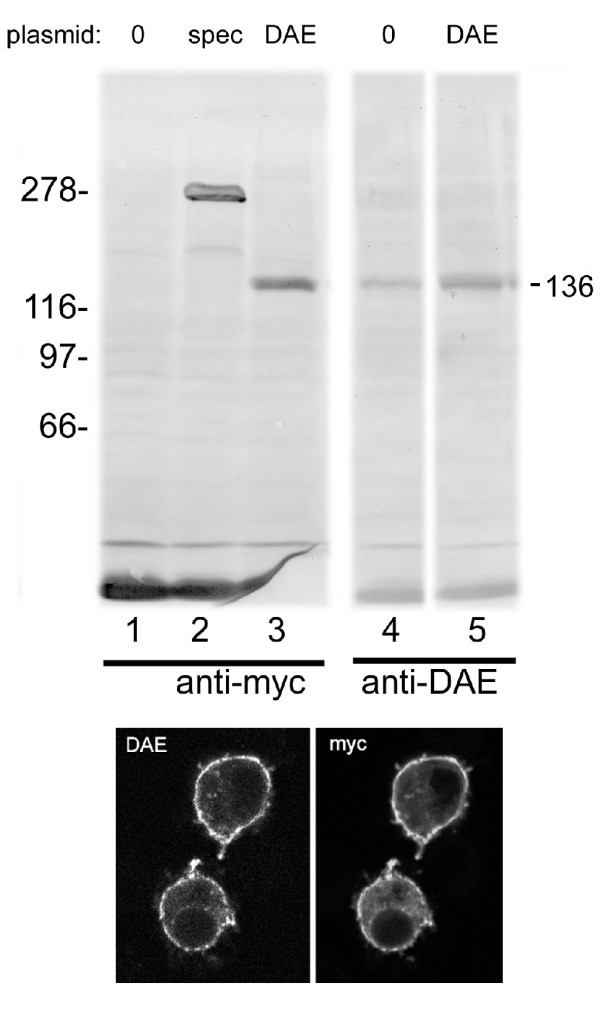
**Expression of endogenous and recombinant anion exchanger in S2 tissue culture cells**. Western blots of total S2 cell proteins were stained with mouse anti-myc epitope antibody (lanes 1-3) or affinity pure rabbit anti-DAE antibody (lanes 4-5). Untransfected cells (0) were compared to transfected cells expressing myc-tagged recombinant β spectrin as a control (lane 2) or myc-tagged recombinant DAE (lanes 3 & 5). The predicted size of the anion exchanger was 136 kD. Transfected S2 cells expressing myc-tagged DAE were stained with the same antibodies and fluorescent secondary antibodies (bottom panels). Staining was primarily observed at the plasma membrane including filopodia.

We engineered a recombinant DAE transgene carrying a myc epitope tag at the N-terminus of the protein. The coding sequence of cDNA RE39419 was used to produce the construct. The purified anti-DAE antibody was used to stain western blots of total proteins from *Drosophila *S2 tissue culture cells. Reactions with control S2 cells detected a faint band with the expected mobility of full-length DAE (~136 kD; Figure 3, lane 4). The relative intensity of the band increased in transfected cells transiently expressing recombinant DAE (lane 5). The same size band was detected with the myc-epitope antibody in transfected cells (lane 3) but not in non-transfected controls (lane 1). A control reaction with transfected cells expressing myc-tagged β spectrin detected a distinct 278 kD band (lane 2).

The same antibodies were used for immunofluorescent staining of S2 cells expressing recombinant myc-tagged DAE. Staining of control cells with the DAE antibody produced very faint plasma membrane staining, close to the threshold of detection (not shown). However, both the myc tag and the DAE antibodies produced strong plasma membrane staining in transfected cells expressing recombinant DAE (Figure 3B). These results establish that the affinity purified DAE antibody detects the expected protein product and that DAE is a plasma membrane protein.

### Localization of DAE in larval tissues

**Figure 4:**
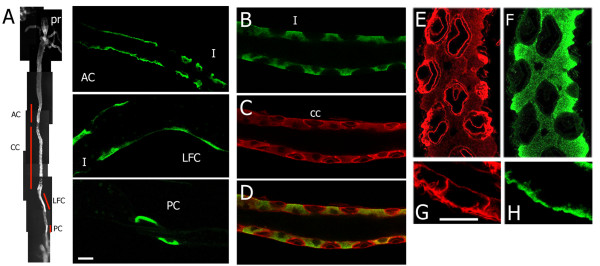
**Immunolocalization of DAE in the larval midgut**. A) Four distinct domains of DAE antibody-labeled cells in larval midgut: anterior cells (AC) upstream of the copper cell domain (CC), large flat cells (LFC) immediately downstream of the CC domain, and 2-3 posterior cells (PC) downstream of the LFC (Pr = proventriculus). The higher magnification views (bar = 10 um) show continuous labeling of the basal membrane of adjacent cells in the AC region (vs. interstitial cells (I) that alternate with copper cells downstream). LFCs were usually visible on only one side of the epithelial tube, downstream of the last interstitial cell. B) DAE staining in the copper cell region revealed a pattern of labeled interstitial cells (I) separated by unlabeled copper cells. C) The copper cells stained with anti-α spectrin antibody appeared as lozenge shapes with relatively bright staining of the basolateral region (merged in D). Higher magnification views of the α spectrin (E) and DAE (F) staining patterns emphasize their lack of overlap. Alpha spectrin was most conspicuous in the basolateral zone of contact between copper cells and interstitial cells and in the banana-shaped apical invagination of copper cells. In contrast, DAE was most conspicuous within the basal cytoplasm of interstitial cells and extended apically in a gradient. G&H) High magnification views of the anterior cells stained for α spectrin (G) or DAE (H) reveals their overlapping distribution in the basal membrane region, but not in the lateral region of cell-cell contact (Bar = 10 um).

The first issue we wished to address with the DAE antibody was its staining pattern in the larval digestive tract, so that we could evaluate its potential involvement in the stomach acid secretion phenotype of α spectrin mutants [27]. Fortuitously, the most prominent region of staining that we observed in larvae was in the midgut (Figure 4A). Within the midgut (Figure 4A), the DAE antibody brightly stained the copper cell region (CC), a cell cluster anterior to the copper cells (AC), the large flat cells (LFC), and a more posterior cluster of 2-3 cells (PC) that may correspond to the iron cells (Figure 4A; [28]). In the anterior cells, DAE staining was confined to the basal surface of the plasma membrane (Figure 4A&4H). In contrast, α spectrin was abundant at lateral sites of cell-cell contact as well as at the basal plasma membrane (Figure 4G). Faint apical staining corresponds to the aβ_H _isoform of spectrin [29]. The large flat cells (LFC) and posterior cells (PC) have an extremely flat morphology that makes it difficult to judge which membrane domain(s) were labeled by the antibody. Staining in the latter two zones was usually limited to cells on only one side of the epithelial tube.

Closer inspection revealed that DAE staining in the copper cell region did not correspond to the copper cells themselves (Figure 4B). Instead, the copper cells were identifiable by their lack of staining with the DAE antibody, and by the bright staining of their apical and basolateral plasma membrane domains with the anti-α spectrin antibody (Figure 4C; merged in D). The DAE signal came from the spool-shaped interstitial cells found in between the copper cells in the middle midgut. Within the interstitial cells, DAE staining formed a gradient that was brightest at the basal surface of the cell and then diminished as it approached the perinuclear cytoplasm near the cell apex. This pattern corresponds to the elaborate infoldings of the basal plasma membrane that are seen by electron microscopy (not shown; [28]), which are most dense in the basal region region of the cell but in some cases extend nearly to the apical surface. A higher magnification view comparing the distribution of DAE (Figure 4F) to α spectrin (E) revealed that α spectrin staining was largely confined to sites of contact between copper cells and interstitial cells and to the banana-shaped apical invagination of the copper cell plasma membrane. The gradient of DAE staining in interstitial cells (Figure 4F) had no counterpart in the α spectrin staining pattern (E).

**Figure 5:**
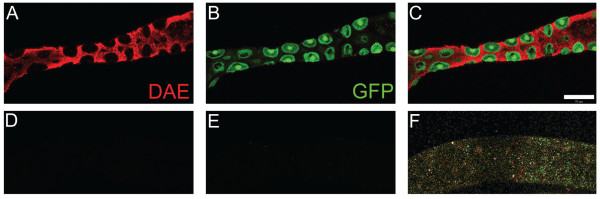
**RNAi knockdown of DAE expression**. The specificity of the DAE antibody was tested by knocking down its expression with RNAi. The midgut-specific Mex-Gal4 driver was used to induce RNAi. In the cross scheme used RNAi-expressing larvae were distinguished from non-expressing siblings by the presence of a GFP reporter in the latter. Larvae were sorted by GFP expression and then dissected and stained with the anti-DAE antibody followed by Texas Red labeled secondary antibody. Control larvae carrying the GFP-marked balancer chromosome exhibited the expected pattern of interstitial cell DAE staining (A) with the GFP reporter fortuitously expressed in neighboring copper cells (B). Siblings that expressed UAS-RNAi (recognized by lack of GFP; E) showed no detectable DAE staining (D), indicating that the antibody was specific for DAE. The merged image (F) was overexposed to demonstrate the presence of the middle midgut. Bar = 20 um.

A control for antibody staining was performed using a UAS-RNAi fly line from the Vienna *Drosophila *RNAi Center (VDRC [ref. [30]]) targeted against DAE. Knockdown of DAE expression was achieved by crossing heterozygous UAS-RNAi parents (UAS-39492/TM3-GFP) to Mex-Gal4, a homozygous line that expresses Gal4 in the larval midgut [31]. Two classes of larval progeny were obtained: a GFP^+ ^control class, where GFP expression indicates the absence of the RNAi-encoding transgene, and a GFP^- ^class that expresses RNAi. Staining of these larvae with anti-DAE antibody revealed a striking reduction in DAE antibody staining in interstitial cells (Figure 5D) relative to the normal pattern in controls (Figure 5A). The midgut DAE knockdown had no detectable effect on larva viability and there was no detectable effect on midgut acidification (not shown) as detected by Bromphenol blue feeding [27]. However, ubiquitous expression of DAE RNAi using tubP-Gal4 [32] produced a lethal phenotype, indicating that there is a critical requirement for DAE function elsewhere in the animal. The lethal RNAi phenotype is consistent with the recessive lethality of a transposable element insertion in the DAE gene that was recently produced by the Berkeley *Drosophila *Genome Project (p[WhY]CG8177^DG29506^; Flybase).

## Discussion

We identified and partially characterized a close relative of the vertebrate SLC4 anion exchangers in *Drosophila *and named it DAE. The amino acid sequence of this protein shares many of the characteristics of other members of this protein family, suggesting that it is likely to mediate Na-independent anion exchange *in vivo*. One major site of expression identified in this study is the stomach acid-secreting region of the larval midgut. We anticipate that there are other important sites of expression given that RNAi-mediated knockdown of DAE expression in the midgut was not lethal whereas ubiquitous knockdown of DAE with RNAi was lethal. Independent confirmation of the essential function of DAE comes from the recent identification of a recessive lethal transposable element insertion in the DAE gene (Flybase).

We previously speculated that a defect in anion exchange activity could account for the stomach acid secretion defect in *Drosophila *α spectrin mutants [27]. This prediction was based on the known interaction of mammalian anion exchangers with ankyrin (and hence spectrin) and the known contribution of anion exchange to acid secretion in mammals. Targeted disruption of mouse AE2 clearly demonstrated an essential role in gastric acid secretion [33]. Deletion of another anion exchanger gene family member (Slc26a9) also blocked gastric acid secretion in the mouse because of its likely effect on chloride secretion [34]. Yet, a knockdown of DAE that was sufficient to eliminate detectable immunoreactivity had no detectable effect on the ability of larvae to produce stomach acid. Thus a different downstream target is likely to be responsible for the observed acid secretion defect in α spectrin mutants.

We set out to characterize DAE with the intent of using it as a membrane marker for the effects of spectrin mutations on interacting membrane proteins. Human erythrocyte AE1 is the best known membrane anchor for ankyrin and spectrin [1]. We conclude from the present evidence that DAE is unlikely to interact with the spectrin cytoskeleton in *Drosophila*. Mammalian membrane proteins that interact with the spectrin cytoskeleton *in vivo *typically colocalize with spectrin and ankyrin by immunofluorescence. Using a sensitive antibody that readily detects spectrin in most *Drosophila *cells, we find that little or no spectrin is expressed in the interstitial cells where DAE is abundantly expressed. If spectrin is present and codistributes with DAE in interstitial cells it is below the threshold of detection of this antibody. Thus, we propose that a spectrin-independent mechanism is likely to explain the peculiar polarized distribution of DAE within basal invaginations of the interstitial cell plasma membrane. In mammalian MDCK and HBE cells, spectrin, ankyrin, the Na, K ATPase, and E-cadherin form a molecular complex and codistribute along lateral sites of cell-cell contact in these polarized epithelial cells [10,35,36]. However, in the anterior cells of the *Drosophila *midgut (AC), in which spectrin and DAE were both expressed, there was no detectable colocalization of the two proteins along lateral contacts between neighboring cells.

Amino acid sequence comparisons also failed to detect conservation of ankyrin-binding activity in DAE. The ankyrin binding site of human erythrocyte AE1 has been mapped to a loop within the N-terminal cytoplasmic domain [25]. Yet, while there was remarkable amino sequence conservation among anion exchangers in regions flanking this loop. (regions R & S in Figure 2), the sequence of the ankyrin-binding loop itself was not conserved in DAE. The flanking sequence conservation is believed to reflect a pH sensing mechanism [24,37], and is not thought to be related to ankyrin-binding activity. There is limited evidence suggesting an interaction between ankyrin and human AE2 and AE3 [38,39]. But sequence comparisons failed to detect conservation of the ankyrin-binding site in these molecules either (not shown). Thus the ankyrin-binding sequence of AE1 may be a unique byproduct of erythrocyte evolution.

The apparent lack of an interaction between DAE and the spectrin cytoskeleton matches a pattern that has emerged in a number of recent studies. As described in the introduction, there are other membrane proteins with ankyrin-binding activity in mammals that do not appear to be conserved in *Drosophila*. We now add DAE to the list, leaving the L1 family cell adhesion molecule neuroglian as the only ankyrin-binding membrane protein whose interaction with ankyrin can be detected in *Drosophila*. What does this apparent lack of conservation mean? One possibility is that many of the known membrane interactions with the spectrin cytoskeleton arose through a physiological need that emerged in the course of vertebrate evolution. Thus, perhaps sodium channels and potassium channels do not require anchorage to the cytoskeleton to carry out their functions in *Drosophila*. Alternatively, it is possible that interactions between the spectrin cytoskeleton and integral membrane proteins are transient in evolution. Perhaps functional links can be swapped between different scaffold proteins such that membrane transporters in *Drosophila *are now linked to cytoskeletal scaffold proteins other than spectrin and ankyrin. Indeed, *Drosophila *spectrin function appears to be redundant in many of the cells that express it (manuscript in preparation), which may be conducive to rapid evolution of protein interactions. If so, it may turn out that different casts of membrane characters are associated with the spectrin cytoskeleton in *Drosophila *and mammals. Further insights into these issues are likely to emerge as the membrane anchors that attach the spectrin cytoskeleton to the plasma membrane in *Drosophila *are discovered and characterized.

## Methods

### Cloning and DNA sequencing

The full-length cDNA RE39419 was obtained from the *Drosophila *Genomics Resource Center. We extended the partial cDNA sequence data available in Flybase to establish that RE39419 belongs to mRNA class B (Figure 1). All sequence analysis was carried out in the DNA services facility at the University of Illinois at Chicago Research Resource Center. All oligonucleotide primers were obtained from Operon. DNA and protein sequence analysis was carried out using GCG software [40]. Amino acid sequence alignments were created using the Gap program. The human anion exchanger accession numbers used were AE1 (X12609), AE2 (NM_003040), and AE3 (NM_005070).

A 420 bp BglII - EagI fragment from the cytoplasmic domain coding region was cloned in the pGEX-3X vector[41] for expression in *E coli *DH5α. Expression studies in *Drosophila *S2 tissue culture cells were carried out using the pWUMB vector [13] which uses the *Drosophila *ubiquitin promoter to drive expression of an N-terminally myc-tagged product. A near-full-length BamHI fragment of the RE39419 cDNA clone was used. An internal BamHI site was inactivated by QuickChange mutagenesis to produce a silent mutation in the coding sequence. Both expression constructs were verified by DNA sequencing of the cloning junctions.

### Production of DAE antibody

Expression of the glutathione transferase fusion protein with the cytoplasmic fragment of DAE was induced by IPTG and the protein was purified by affinity chromatography. Rabbits were immunized by popliteal lymph node injection of 50 ug purified antigen, followed by 50 ug antigen subdermally at one month intervals. Rabbits were pre-screened for lack of preimmune reactivity and they showed a marked response to the antigen by the second boost (not shown). Immune serum was affinity purified and antibody was counter-adsorbed with purified glutathione transferase for all of the studies described here.

### Expression in S2 cells, western blots, and confocal microscopy

Transfection of S2 cells was carried out using lipofectamine (Invitrogen). Cells were processed for staining as previously described [9] using mouse monoclonal 9E10 (anti-myc; [ref. [42]]) and the affinity purified rabbit anti-DAE followed by Texas Red anti-mouse and FITC anti-rabbit secondary antibodies (Zymed Laboratories). Western blots were produced using standard methods, stained using the above antibodies with alkaline phosphatase-coupled secondary, and developed using BCIP and NBT (also from Zymed). Stained cells were viewed and photographed using an Olympus FV500 confocal microscope using a 60× plan-apo oil-immersion objective (N.A. 1.4) and Fluoview 2.1 software. Brightness settings were optimized for saturation using the photomultiplier and settings were kept constant in comparisons of transfected and untransfected cells.

## Competing interests

The authors declare that they have no competing interests.

## Authors' contributions

RRD conceived the study, designed the experiments, helped with data acquisition and analysis and wrote the manuscript. All authors read and approved the manuscript.

AD performed transfections, produced full length expression plasmid and performed localization experiments.

CB produced bacterial expression constructs and produced the antibody and performed DNA sequence analysis.

GHM performed RNAi experiment and localization of DAE.
